# The impact of long COVID on UK healthcare workers and their workplace: a qualitative study of healthcare workers with long COVID, their families, colleagues and managers

**DOI:** 10.1186/s12913-025-12677-x

**Published:** 2025-04-09

**Authors:** Amani Al-Oraibi, Carolyn Tarrant, Katherine Woolf, Laura B. Nellums, Manish Pareek

**Affiliations:** 1 Department of Respiratory Sciences, University of Leicester, Leicester, UK; 2 Development Centre for Population Health, University of Leicester, Leicester, UK; 3 Population and Lifespan Sciences, School of Medicine, University of Nottingham, Nottingham, UK; 4 Department of Population Health Sciences, University of Leicester, Leicester, UK; 5 University College London Medical School, London, UK; 6 College of Population Health, University of New Mexico, Albuquerque, NM USA; 7 NIHR Leicester Biomedical Research Centre, Leicester, UK; 8 Department of Infection and HIV Medicine, University Hospitals of Leicester NHS Trust, Leicester, UK; 9 NIHR Applied Research Collaboration East Midlands, Leicester, UK

**Keywords:** Long COVID, Healthcare workers, Qualitative research, Workplace support, Cultural diversity, Workforce management, NHS, Occupational health, Support networks, Healthcare managers

## Abstract

**Background:**

Healthcare workers (HCWs) have been particularly impacted by long COVID, with negative effects on their work patterns and wellbeing. The aim of this study was to explore the intersection between work and long COVID for HCWs, to understand the impact of long COVID on their professional identify, their orientation to work, their wellbeing as professionals, and support needs and strategies for them as well as their managers to continue to work.

**Methods:**

This qualitative study was conducted through semi-structured online interviews with three groups: HCWs with long COVID, their support network members, and healthcare managers between March 2023 and May 2024. To maintain confidentiality and address concerns about workplace stigma, healthcare managers were not matched with specific HCWs. Participants were recruited through purposive and snowball sampling, until data saturation was reached, defined as the point at which no new insights or themes were identified. Data were analysed using reflexive thematic analysis.

**Results:**

A total of 42 participants were interviewed from three groups, comprising 24 HCWs, five support network members, and 13 healthcare managers. Four key themes were identified describing experiences of long COVID for HCWs: (1) Living and coping with long COVID as a HCW, (2) Workplace impact and adjustments, (3) The uncertain nature of long COVID and challenges of the definition, and (4) Feelings of guilt, stigma and blame.

**Conclusion:**

In conclusion, long COVID has created significant challenges not only for HCWs but also for their managers, who struggled with staffing shortages and lack of clear guidance, and support network members who experienced emotional strain while providing care. The combination of these challenges threatens NHS workforce stability and service delivery. Developing and embedding flexible, standardised workplace interventions—such as phased return-to-work policies and tailored occupational health support—could mitigate these impacts and inform scalable solutions across diverse healthcare systems. Enhanced training for healthcare managers and further research into culturally diverse coping mechanisms could improve support for affected HCWs, reduce stigma, and contribute to a more stable and resilient healthcare workforce. While based in the UK, these findings offer important insights for health systems globally that are grappling with the long-term workforce implications of long COVID.

**Supplementary Information:**

The online version contains supplementary material available at 10.1186/s12913-025-12677-x.

## Introduction

Long COVID, characterised by signs and symptoms that continue or develop after acute COVID-19 infection for ≥ 12 weeks [1], continues to affect individuals worldwide with varying severity, duration and experiences [2, 3]. Several qualitative studies have highlighted how individuals with long COVID experience changes in their self-identity and face challenges with healthcare services [4]. A recurring theme in existing qualitative research is the general lack of understanding and support regarding long COVID, including about healthcare providers [5, 6]. For many participants, this led to frustration and exacerbated feelings of illness uncertainty, leading individuals to resort to self-management of their symptoms [6]. This uncertainty was reflected in encounters with family, friends, employers and healthcare professionals from whom care was sought [6]. 

Long COVID has caused significant reductions in physical and mental health among HCWs compared to their pre-infection health [7]. A mixed-methods study exploring the impact of long COVID on UK National Health Service (NHS) HCWs found that 90% of surveyed HCWs experienced activity limitations due to long COVID, with many reporting symptoms such as fatigue and cognitive impairments [8]. Two-thirds of participants had taken sick leave, and a notable proportion reported permanent changes to their work responsibilities as a result of their ongoing symptoms [8]. Interviews with HCWs have highlighted dissatisfaction with the support received from employers, despite some positive examples of workplace adjustments, such as flexible working hours and phased returns [8]. In another study in which they conducted 13 interviews with doctors experiencing persistent symptoms revealed feelings of distress, particularly stemming from a sense of being ‘let down’ by their own doctors and the NHS, with many perceiving that they were not believed [9]. These narratives highlighted a moral dilemma: while they wished to be treated as ‘patients,’ they also felt professionally dissatisfied with the care they received [9]. A longitudinal qualitative study was conducted in the UK and explored the healthcare experiences of HCWs and patients with long COVID, revealing significant concerns about the lack of clear diagnostic criteria, treatment pathways, and occupational support among participants [10]. 

Long COVID has resulted in substantial work absences and modifications to work patterns [11, 12]. Evidence shows that perceived work ability among HCWs decreased compared to a time before they became infected by COVID-19 [13]. 

In the UK, a study found that 18% of doctors with long COVID had not returned to work due to illness, while 40% of those who had returned to work required a phased return or amended duties [14]. Another UK-based study found that 4.9% of those who had long COVID required a period of additional sick leave following their initial isolation period [15]. 

Existing research has focused predominantly on the physical and psychological impact of long COVID on HCWs using quantitative methodologies, while qualitative studies have primarily explored HCWs’ experiences of long COVID symptoms rather than its impact on their professional lives. Therefore, we aimed to explore the intersection between work and long COVID for HCWs, to understand the impact of long COVID on their professional identify, their orientation to work, their wellbeing as professionals, and support needs and strategies for them as well as their managers to continue to work.

## Methods

This study adhered to Consolidated Criteria for Reporting Qualitative Research (COREQ) guidelines for reporting qualitative research [16] (see Supplementary material 1 in supplementary materials).

### Participants

Three groups of participants were interviewed:

1. HCWs with previous COVID-19 infection and long COVID: to explore their experiences with long COVID and the impact of long COVID on their work.
2. HCW support network members: to explore in-depth their experiences of supporting HCWs with long COVID, including the impact on HCWs’ home and work lives.
3. HCW managers and NHS Human Resources (HR) managers: to gain their perspective on sick leave policies and the impact of long COVID on staff management, which introduced insights distinct from the experiences shared by frontline HCWs. Additionally, to explore how they perceive and navigate the challenges of supporting HCWs with long COVID while ensuring the quality of patient service delivery. To maintain confidentiality and address concerns about workplace stigma, healthcare managers were not matched with specific HCWs.

### Sampling strategy, and recruitment

Participants were sampled sequentially in three stages:

1. HCWs with previous COVID-19 infection and long COVID were recruited by email to the United Kingdom Research study into Ethnicity and COVID-19 outcomes among healthcare workers (UK-REACH) qualitative study cohort [17], between March and August 2023 using purposive sampling, guided by UK census, taking into account ethnic background, gender, job role, and age until data saturation was reached.

UK-REACH is a mixed-method nationwide study initiated to provide rapid evidence on COVID-19 outcomes among HCWs and inform national policy [18]. The qualitative component of study was conducted between December 2020 and July 2021 where 164 HCWs were interviewed including workers from various staff grades, job roles, age, sex, ethnicity, migration status, and United Kingdom nation [17].

(2) Support network members of interviewed HCWs were sampled using snowball sampling between September and December 2023. They were recruited by inviting HCW participants to tell their support network (family members, friends and healthcare colleagues) about the research If they wished, participants contacted their support network member(s) to get permission to share their contact details with the research team, who then invited them to take part.
(3) Healthcare managers and Human Resources (HR) managers were purposively sampled through advertisements on social media, -UK-based conferences that involved HCWs and via the project Stakeholder Group (STAG) between December 2023 and May 2024.

UK-REACH has worked closely with national and local organisations in designing the study [18]. They set-up a Patient and Public Involvement and Engagement (PPIE) and a STAG who met regularly and directly inform the design of materials, study methodology (recruitment) and analysis [18]. 

### Procedure

All interviews were conducted by AAO in 2023 and 2024 virtually using Microsoft Teams. All interviews were conducted online due to the diverse geographical spread and demanding work schedules of HCWs, which made in-person interviews impractical. Conducting interviews virtually allowed for greater flexibility and accessibility, and was deemed appropriate given participants’ high levels of digital literacy. Good qualitative practice was followed throughout, including steps —such as beginning with informal conversation, ensuring confidentiality, and using open-ended and follow-up questions to encourage comfort and detailed responses in the virtual environment. Eligible participants were provided with a Participant Information Sheet (PIS) prior to joining the study and written informed consent gained via email.

Interview topic guides were developed, with inputs from the UK-REACH’s PPIE and STAG groups. The HCW topic guide explored the impact of acute and long COVID on their work, home lives, physical health, and mental wellbeing, their coping mechanisms, and the workplace support they received. Questions for support network members focused on the impact of the HCWs’ long COVID symptoms emotionally, financially, and in terms of home and work responsibilities. Healthcare and HR managers were asked about their experiences of managing teams that included HCWs with long COVID, organisational policies, workload management, and the emotional burden of supporting HCWs.

Interview topic guides used in our interviews are shown in Supplementary Material 2.

### Data analysis

Interview recordings were transcribed verbatim and anonymised by a professional transcriber. AAO analysed all data using reflexive thematic analysis [19]. 

Open coding was conducted for the initial few transcripts across the three participant groups which then were merged to develop a coding framework using NVivo (version 1.6.1) [20]. Initially, transcripts from each of the three participant groups were coded separately by AAO to identify group-specific codes. After this initial coding phase, codes that overlapped conceptually were merged to form an overarching coding framework into higher order themes, integrating findings across groups by examining the codes that emerged from each group’s transcripts, identifying commonalities, and comparing and contrasting the codes across the three groups to ensure integration of perspectives. Preliminary themes were reviewed and refined by CT, LBN, KW and MP, ensuring that the perspectives of PPIE and STAG groups were incorporated. Revisions were made based on their suggestions, with final themes generated using techniques such as narrative summaries and mind maps to describe themes and the relationships between them.

### Patient and public involvement and engagement (PPIE)

In addition to UK-REACH study PPIE and STAG members, using social media platforms (e.g., Facebook, Twitter-X, and UK-REACH study website), we recruited into PPIE additional six HCWs with long COVID from different occupational roles (both clinical and non-clinical) and ethnic backgrounds. This was to inform and support this research study by bringing in their unique insights and perspectives, including their lived experiences with long COVID and how COVID-19 has impacted their home and work lives in the long term. We had four biannual PPIE and STAG group meetings. They helped shape the topic guide and refine the preliminary themes.

### Reflexive statement

The research team comprises individuals from diverse demographic backgrounds, including ethnicity (Arab, South Asian, White), migration status (migrants and UK-born), and gender (both male and female). Their professional and academic expertise includes public health, social work, education, anthropology, pharmacy, and health policy. Throughout the process of data analysis and coding, team members-maintained reflexivity to ensure that the voices of HCWs from different ethnic and professional backgrounds were authentically represented and that the findings could lead to meaningful support for HCWs with long COVID.

## Results

### Participant characteristics

In total 42 participants were interviewed, comprising 24 (57%) HCWs who have or have had long COVID, 13 (31%) healthcare managers who lead teams of HCWs with long COVID, and 5 (12%) support network members of HCWs with long COVID.

The highest percentage of participants within each group fell in the following age ranges: HCWs (55–64 years; 33%), healthcare managers (45–54 years; 54%), and support network members (45–54 years; 60%).

Among HCWs who provided their gender 66% (*N* = 27) were female, and half of the total number of participants (50%, *N* = 21) were from ethnic minority backgrounds. Demographic characteristics of participants are presented in Table 1.

**Table 1:** A summary of participants’ demographic characteristics

Category	Description	Healthcare workers (*N* = 24)	Healthcare managers (*N* = 13)	Support network members (*N* = 5)
**Gender**	**Female**	16 (66.7%)	7 (53.8%)	4 (80.0%)
	**Male**	8 (33.3%)	6 (46.2%)	1 (20.0%)
**Age Range (years)**	**25–34**	-	1 (7.7%)	-
	**35–44**	4 (16.7%)	2 (15.4%)	1 (20.0%)
	**45–54**	7 (29.2%)	7 (53.8%)	3 (60.0%)
	**55–64**	8 (33.3%)	2 (15.4%)	1 (20.0%)
	**65–74**	1 (4.2%)	-	-
**Ethnicity**	**British White**	9 (37.5%)	5 (38.5%)	-
	**Other White Background**	2 (8.3%)	-	-
	**British Asian**	2 (8.3%)	-	-
	**Indian**	4 (16.7%)	2 (15.4%)	-
	**Black African**	-	1 (7.7%)	-
	**Other Asian**	-	3 (23.1%)	-
	**Mixed**	1 (4.2%)	-	-
	**Other**	2 (8.3%)	1 (7.7%)	-
	**Prefer not to say**	1 (4.2%)	-	-
**Job Role**	**Medical**	3 (12.5%)	-	-
	**Allied Health Professionals**	8 (33.3%)	-	-
	**Nursing**	6 (25.0%)	-	-
	**Healthcare Science**	5 (20.8%)	-	-
	**Administration**,** Management & Support**	5 (20.8%)	-	-
	**Clinical Management**	-	4 (30.8%)	-
	**Administrative Leadership**	-	4 (30.8%)	-
	**Specialist Medical Leadership**	-	4 (30.8%)	-
	**Scientific Leadership**	-	1 (7.7%)	-
**Relationship with HCW**	**Live in the same household (partner)**	-	-	2 (40.0%)
	**A friend/Neighbour**	-	-	1 (20.0%)
	**A work colleague**	-	-	2 (40.0%)
**Country of Birth**	**UK**	16 (66.7%)	5 (38.5%)	-
	**Outside UK**	7 (29.2%)	8 (61.5%)	-
**Vaccination Status**	**Have had four doses**	10 (41.7%)	-	-
	**Have had three doses or fewer**	15 (62.5%)	-	-

(-) indicates that the question was either not asked in the demographics form or the participant did not provide an answer

**Fig. 1:**
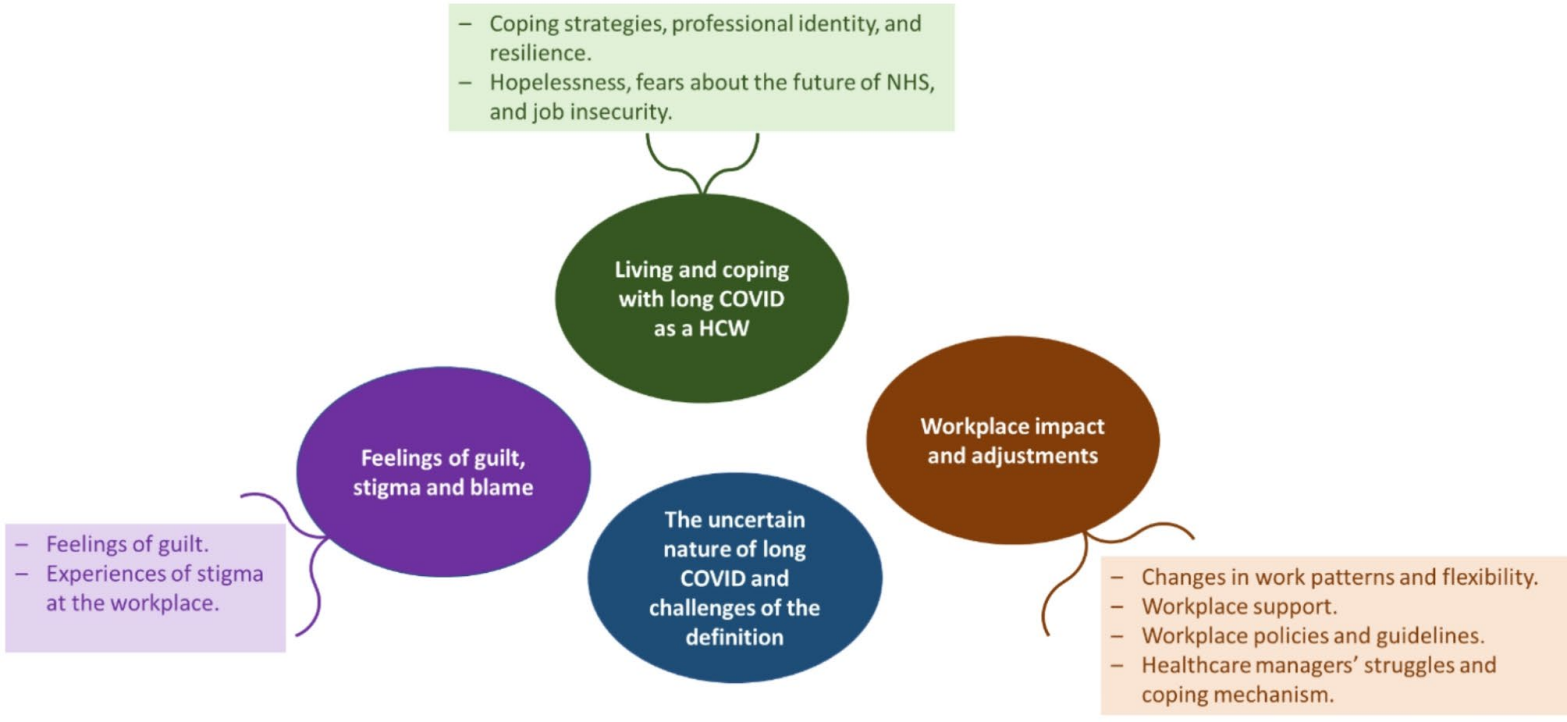
Identified themes and subthemes

Four key themes and eight sub-themes were identified from the combined data: (1) Living and coping with long COVID, (2) Workplace impact and adjustments, (3) Impacts of perceptions of long COVID and challenges of the definition, and (4) Feelings of guilt, stigma and blame as shown in Fig. 1.

### Theme 1: living and coping with long COVID as a HCW

For HCWs with long COVID, professional identities and professional norms meant they felt obliged to try to continue working despite significant symptoms. Wider concerns about the impact of COVID-19 on the NHS placed additional pressures on HCWs to try to continue to work, but also resulted in additional anxieties and emotional strain.

#### Subtheme 1.1: coping strategies, professional identity, and resilience

Despite HCWs’ efforts to manage their symptoms, their professional identity and sense of duty often became central to their resilience, pushing them to “carry on” even when their health demanded otherwise.

*“I haven’t got any choice. You just have to get on with it. You’ve just got to really focus and really concentrate and just slow down a bit. Nothing else you can do.” (HCW02, Female, Pharmacist, White British)*

Cultural norms also influenced coping mechanisms. One participant avoided using sick leave, citing their cultural background and professional ethics as reasons to continue working despite significant symptoms.

*“So the way I look at it is I’m [ethnic minority background] who’s been here [>10 years]. So when I look at it, what would I do if I was back in [home country]?…I go and I make sure that I’m, I try and protect myself and then I carry on with it. I’m not going to sit and cry over it… I have not called in sick in the last fifteen years. I’m not going to call in sick now just because there is a loophole in the system.” (HCW17, Male, Manager, Asian)*

These coping strategies often came at a cost, as participants prioritised their work and family over their own recovery.

HCWs frequently described being unable to manage daily household tasks or care for their families due to persistent symptoms, placing additional responsibilities on their support networks and creating emotional strain within their households.

*“Well I was a bit worried because I didn’t know whether he was going to ever go back to being his normal self because he was so debilitated at first by it, you know, and I think it affected me because it was worrying him a bit.” (SN01, a HCW’s wife)*

#### Subtheme 1.2: hopelessness, fears about the future of NHS, and job insecurity

Long COVID symptoms, combined with pressures to return to work, created significant stress for HCWs. Many felt compelled to return prematurely, fearing job loss or being perceived as unreliable by colleagues and managers. This fear was compounded by the unpredictable nature of their condition, leaving HCWs uncertain about their future professional capacity. For some, the persistent nature of their symptoms led to a sense of despair about the future.

*“I’m now back at work and struggling hugely with it because I still have long COVID and I see them [managers] two mornings, one face to face – no one at home – I’m sorry I’m going to cry!” (HCW13, Male, Manager, White British)*

Furthermore, many expressed deep concerns about the future of the NHS. As frontline workers, they were aware of the strain placed on the system during the pandemic, and several feared that the NHS would be unable to recover from the lasting damage. One participant reflected on the loss of colleagues to COVID-19 and the subsequent staffing shortages, worrying that without significant investment, the NHS might not be able to address the backlog of patients and the increased demand for services. This fear of collapse added to their mental strain, as they not only worried about their health but also about the ability of the healthcare system to support them and others in the future. This led many HCWs to feel a profound sense of hopelessness about their situation. For some, the emotional impacts of these combined worries became overwhelming.

*“It would be nice to have somebody to wave a magic wand and go “We’ve come up with something to cure long Covid”. That would be amazing, but it’s not going to happen, certainly not in my working lifetime.” (HCW02, Female, Pharmacist, White British)*

### Theme 2: workplace impact, and adjustments

Long COVID disrupted work patterns and family life for HCWs; flexible working arrangements and workplace support were vital, but this need for flexibility and support could be difficult to accommodate, creating challenges for managers and additional burden for colleagues. A lack of clear, long COVID-specific policies left manager feeling unprepared to support their teams.

#### Subtheme 2.1: changes in work patterns and flexibility

Many HCWs spoke about the challenges they faced having to reduce their working hours or transition less physically demanding roles to accommodate their ongoing or persistent symptoms. This was particularly challenging because of the pressures at their workplace post-COVID-19 describing it as a “backlog”, where they felt that the NHS was unable to meet their demands. In addition to workplace adjustments, the disruption caused by long COVID extended beyond work to the home.

The introduction of flexible working arrangements was a positive change for some HCWs, allowing them to manage their symptoms while continuing to contribute professionally. Remote work was particularly beneficial for those struggling with fatigue, as it eliminated the need for commuting and allowed for more flexibility in how they structured their day. However, for others, the shift to remote work came with challenges, particularly around feelings of isolation and missing the social aspect of working in a team environment.

*“Working from home and having flexible working patterns, maybe people working reduced hours, compressed hours, that kind of thing is much more commonplace now particularly within our department”. (HCW06, Female, Pharmacist, Asian)*

#### Subtheme 2.2: workplace support

Workplace support experiences varied for participants. Some HCWs described positive experiences, where their managers and colleagues were understanding of their condition and worked with them to make necessary adjustments. For instance, they expressed appreciation of how their manager allowed a phased return to work, starting with just a few hours a week and gradually increasing over time.

*“I was always told that if I needed longer, that was absolutely fine and not to come back until I was a hundred percent better and to look after myself…Yes, so supportive”. (HCW24, Female, Midwife, White British)*

On the other hand, others faced significant challenges in accessing the support they needed. Some HCWs encountered difficulties from HR departments when requesting accommodations such as phased returns or flexible work hours. In certain cases, participants expressed their frustrations with occupational health services, noting that the process was often slow and unresponsive, leaving them without adequate support for long periods. There was also a mention of how they needed to spend time and efforts to explain to their management their unique experiences/symptoms with long COVID to be understood. This was also mentioned by support network members (work colleagues) in which they voiced frustrations with the lack of formal support from HR for their colleagues, which extended the strain on the team. Delays in implementing necessary accommodations, such as working from home, left both participants and their support networks feeling unsupported and let down by the system.

*“If I’m being completely blunt I don’t think the Trust has really done enough about it, to look into it and maybe – they’ve done the whole work from home stuff which I guess could be seen as maybe helping out with people who might have long Covid but really it feels like they’re putting a Band-Aid over it”. (HCW01, Male, Clinical Scientist, White British)*

*“She requested to work at home on the middle day… but it wasn’t recognised that she’d got this condition and that she was really struggling. So I don’t think they were very supportive with her when she came back.” (SN02, a HCW’s work colleague)*

From the perspective of managers, many expressed that while they worked hard to accommodate HCWs with long COVID, this often placed a considerable emotional and logistical strain on them. Adjusting workloads, reorganising staff, and ensuring patient care while maintaining fairness across the team required significant effort. Managers also noted that in making these adjustments, they sometimes had to compromise their own time and balance between professional and personal responsibilities.

*“I compromised my family a bit. Because I felt I need to lead by example. If I’m asking the remaining of the staff to push harder, I need to push harder than them…It’s hard”. (MNG02, Female, Manager, Asian)*

Despite the pressures, many managers demonstrated a commitment to supporting their staff’s health and wellbeing, emphasising the importance of recovery over work. Nevertheless, managers often described a sense of being caught in a dilemma—wanting to support their staff but feeling as though they were neglecting others in the process. This created a complex and emotionally challenging situation for them.

*“I was very upfront in telling them ‘if you don’t feel well don’t even think of come in because you need to look after yourself first of all…’. It’s a dilemma for me because I felt when I’m supporting one I’m neglecting the other one, that’s how it felt for me as a manager, but then you’re stuck between a rock and a hard place.” (MNG04, Female, Lead Nurse, Other ethnic group)*

Colleagues of participants played a big role in providing day-to-day assistance and support at workplace. Some support network members described making extra efforts to check in on their colleagues and assist with their workloads. However, balancing being supportive while also ensuring the department ran smoothly presented challenges.

For some, the extended absence of their colleagues and the additional workload was stressful Although they tried to manage the extra responsibilities, it often left them feeling overwhelmed, and in some cases, close to burnout. Despite these challenges, most support network members expressed empathy towards their colleagues’ struggles, recognising that their colleagues felt guilty about the added burden they created.

*“I think as a team, we’ve been very supportive of our colleague and checking in with her. I think what’s been very difficult is there’s the fatigue problem when she was off and how she managed the fatigue when she came back and just her resilience with fatigue now is obviously challenging for her. And for her to work in the role now she’s back, it’s finding that balance of being supportive but also running the department.” (SN05, a HCW’s work colleague)*

#### Subtheme 2.3: workplace policies and guidelines

Managers expressed feelings of indecision, fearfulness of not providing enough support for HCWs, and uncertainty regarding the policies and guidelines in place to support staff members with long COVID. Reliance on general sickness policies proved insufficient for managing a complex condition like long COVID, which required more specific guidance. Many managers described the lack of clear, long COVID-specific policies as leaving them unprepared and anxious about adequately supporting their teams.

*“I’ve been fearful, I’m fearful that I may not be addressing their needs…will I be saying things that is quite something that may offend them? it’s like walking on eggshells because there’s no – to begin with there’s no policy to help you to guide you with your conversations… Long Covid is such a new ailment that even the specialist doesn’t fully understand as yet.” (MNG04, Female, Lead Nurse, Other ethnic group)*

Managers frequently felt that long COVID was treated like other health conditions, without consideration of its unique nature, leading to ambiguity about how best to support staff.

*“I feel that with Long Covid it’s either we need a very clear direction… so that the resentment doesn’t boil – I think it’s the boiling point for the longest time because of the lack of clarity, of the lack of support” (MNG03, Female, Senior Matron, Other Asian)*

Additionally, the absence of proper guidance left managers relying on personal assumptions, which negatively affected how they perceived long COVID cases.

*“We weren’t given any guidance …So when I had these discussions I thought oh my god, I didn’t know it’s that bad, because I had Covid myself, so you base it on how you felt and having had Covid myself, you will not automatically switch on that Long Covid thinking because you feel that they’re just making it up”. (MNG03, Female, Senior Matron, Other Asian)*

Managers also spoke about the difficulty of keeping up with constantly changing policies, which made them feel extra pressure. The frequent updates made it challenging to provide consistent and informed support to their staff.

*“We had a protocol in place but it was a document that they kept changing every two weeks, so it was hard to keep track of ‘OK what shall we do now’, by the time you learn that protocol it was changed again.” (MNG10, Male, Senior Pharmacist, Black African)*

Many managers expressed a desire for comprehensive training to better handle these situations. While leadership programmes included general HR policies, they felt this lacked depth on managing long COVID.

*“You don’t receive training about the policy but you receive training as a clinical lead. So I’ve just finished, in January I finished leadership training with the Trust… So in that session it tells you about what to do when you have somebody who’s ill or somebody who’s pregnant or somebody who has different needs.” (MNG13, Female, A manager doctor, Asian)*

#### Subtheme 2.4: healthcare managers’ struggles and coping mechanism

Healthcare managers faced significant challenges due to due to prolonged staff absences from long COVID, leading to increased workloads, stress, and burnout.

*“Five members of staff had prolonged sickness… it’s staggered kind of sickness, but they were off sick continuously for a good year, some of them two years, so the staffing has been affected, obviously the morale has been affected”. (MNG03, Female, Senior Matron, Other Asian)*

These long absences disrupted team dynamics and reduced morale, further compounding the difficulty of maintaining normal operations. Additionally, the struggle to cover shifts, especially with repeated absences, meant that the workload became overwhelming for those remaining, leading to a decline in service delivery.

*“If then you start to lose like two Band 6s*^1^* on one shift, you’re struggling to cover your areas... so it definitely has an impact on the way the department runs, the patient care and the standards of care” (MNG07, Female, Sister, White British)*

Managers had to find creative solutions, often without additional funding, which added to their stress. Phased returns to work, while beneficial for recovery, created financial challenges.

*“We are constantly trying to find more creative ways of dealing with the workload.” (MNG12, Male, manager doctor, Arab)*

*“We do a phased return… but that impacts on the budget too because they’ll be paid as fulltime and then we cover their bank shift. We’re double paying” (MNG02, Female, Manager, Other Asian)*

Some managers reconsidered their roles due to the relentless pressure of managing long COVID-related issues.

*“It’s made me consider taking a different job... because I think sometimes, especially things like Long Covid, it just cause a lot of headaches that we didn’t have solutions to”. (MNG10, Male, manager pharmacist, Black African)*

Despite the challenges, some managers noted positive outcomes from the experience and fostered a sense of accomplishment for some. They felt that managing the complexities of long COVID helped them grow stronger in their roles, improving their problem-solving skills and resilience

*“It makes you stronger because you take the department through something which is really difficult… it works nicely for your people skills, it works nicely for increasing your resilience” (MNG13, Female, manager doctor, Asian)*

*I’ve learned to be really flexible now with policies, I've learned how to tailor my advice more to individual staff, I'm now more aware of the support networks that are available in the hospital.” (MNG10, Male, Senior Pharmacist, Black African)*

### Theme 3: the uncertain nature of long COVID and challenges of the definition

Participants expressed varied perceptions of long COVID, reflecting personal experiences and societal attitudes. While some acknowledged long COVID as a significant condition, others demonstrated scepticism, particularly in cases where symptoms were difficult to measure or validate. These perceptions had direct implications for workplace adjustments and support.

*“I think it might be the same thing with Long Covid… there probably will be people out there who are using it as a way of avoiding work or just avoiding things within a family”. (HCW13, Male, Manager clinician, White British)*

HCWs and managers alike experienced significant challenges due to the lack of a well-defined, formal understanding of long COVID. Many HCWs expressed frustration over the difficulty in proving the condition since it is largely self-reported without any documented evidence of initial COVID-19 infection. Managers also spoke about facing challenges in handling long COVID due to its variability in terms of symptoms and recovery times that made it difficult to plan and adjust workloads. They explained that this unpredictability not only affected service delivery but also placed a strain on the remaining workforce, who often had to work longer hours to cover for absent colleagues. This unpredictability extended to the return-to-work process, creating feelings of uncertainty for them as leaders on what to expect from HCWs affected when they return to work. Furthermore, the lack of clear guidelines or understanding of long COVID exacerbated this problem as it felt to them as a “guesswork”.

*“I do know that there is a huge issue now about the fact that it’s difficult to prove as it were that you’ve got long Covid because by definition it’s self-reported.” (HCW08, Female, Dietician, White British)*

Moreover, the lack of formal recognition of long COVID by health authorities led to informal pressures for staff to return to work prematurely. This was seen as a major issue. Managers felt that there was a cycle of returning too early only to relapse which was a real a challenge for managers trying to balance compassion for affected staff with the demands of maintaining service delivery.

*“People came back a bit too early, started working... and they went off sick again.” (MNG10, Male, Senior Pharmacist, Black African)*

### Theme 4: feelings of guilt, stigma and blame

HCWs with long COVID experienced profound feelings of guilt due to their prolonged absence, reduced capacity to work, and the additional burden placed on colleagues. Many felt a strong sense of personal responsibility to continue working despite debilitating symptoms. Managers recognised this internalised guilt among their staff and attempted to reassure them, but HCWs often struggled with the pressure of wanting to contribute while physically being unable to do so.

#### Subtheme 4.1: feelings of guilt

HCWs dealing with long COVID reported feelings of guilt, which was recognised by managers. Managers noticed that staff members who were experiencing long COVID often felt guilty about their prolonged absence or reduced ability to work, even when such feelings were not explicitly imposed by their workplace. Despite reassurance from managers, employees still carried a personal burden, feeling the need to explain and justify their inability to return to work. This guilt was compounded by the invisible nature of long COVID symptoms, such as fatigue and brain fog, which made some employees feel misunderstood. These feelings of guilt were not necessarily a result of direct pressure from their workplace but were self-imposed by employees who were used to being high-performing, particularly in healthcare roles.

*“She expressed feeling guilty because she herself didn’t know what’s going on, so because she doesn’t know what’s going on she doesn’t know how long she can be off sick for. So from my point of view, it was more reassuring her that it’s fine to take that time off, we’re here to help and what we need is for her to at least keep us updated on how she’s getting on”. (MNG10, Male, Senior Pharmacist, Black African)*

HCWs themselves also expressed feelings of guilt. Many HCW expressed the internal conflict of wanting to contribute more at work but physically being unable to. This pressure to continue working often came from within, as these employees did not want to let their colleagues down, despite not being fully capable of performing their duties. Other HCWs described the frustration and guilt of having to take sick leave, explaining how difficult it is in healthcare to step away from work. This internal conflict of prioritising their health while feeling responsible for patient care and the strain it puts on their colleagues was a recurring theme. These feelings of guilt were often exacerbated by the pressure to justify the time off and their inability to return to work. The pressure was not necessarily from management, but rather a personal sense of responsibility, particularly in a context where understaffing and the impact on patient care were constant concerns.

*“I feel I push myself because I want to contribute… I don’t want to feel like a complete drain on them” (HCW16, Female, Dietician, other ethnic group)*

*“It’s really, really hard in healthcare to not go in… the guilt, and knowing the expense, and trying to get somebody to cover”. (HCW02, Female, Pharmacist, White British)*

*“You’re seen as weak, you’re seen as somebody who doesn’t really sort of care, somebody who is letting the team down, you know, it’s short staffed and you’re letting your colleagues down. You do feel quite a lot of pressure to continue working in the areas that are short staffed. And certainly this idea of letting your colleagues down is hard.” (HCW05, Female, Nurse, British Asian)*

Support network members also expressed feelings of guilt as they felt that the support they give was not enough and were feeling bad for not doing more.

*“It made me, I mean it made me feel bad for her because I couldn’t do any more. Yeah, I just text or call. That’s all I could do. I did everything I possibly could.” (SN04, a HCWs’ neighbour)*

#### Subtheme 4.2: experiences of stigma at the workplace

HCWs and managers discussed the stigma surrounding long COVID in the workplace. For HCWs, the experience of stigma often came from a sense of being judged by work colleagues. Although many HCWs reported supportive environments, with colleagues who were understanding, others faced scepticism or negative attitude, leading to a perception by managers that they were being seen as *“workshy or opportunistic”*. This scepticism created an environment where HCWs felt stigmatised for taking time off to recover from long COVID, even if they were genuinely unwell. They felts the pressure to justify their illness and convince their colleagues of its existence created further pressure on them as they felt they were judged hardly at the workplace. In some cases, the stigma was subtle, manifesting as gossip or cynical remarks about whether the illness was legitimate. Additionally, some HCWs felt the need to justify their absence due to long COVID to avoid being seen as faking or exaggerating their symptoms.

*“there is a huge stigma attached to people that go off sick long term for COVID, unless there is really overwhelming evidence that there’s something badly wrong with them” (HCW05, Female, Nurse, British Asian)*

*“there was some scepticism… people thought maybe they were playing it up a bit” (HCW09, Female, GP, White British)*

For managers, the stigma towards long COVID employees was coupled with managing the frustration of other colleagues who had to take-on the increased workload. Managers observed that the resentment toward staff members with long COVID was not necessarily personal, but rather a response to the strain placed on the team. This type of resentment, while not always openly hostile, created some tension, with employees feeling unwelcome or blamed for being unable to contribute fully, according to managers. In some cases, this stigma led to long COVID employees feeling defensive upon their return to work, as they sensed their colleagues’ frustration with having had to cover their duties. The absence of a clear understanding of long COVID only worsened the situation, as employees and managers alike struggled with the unpredictable nature of the illness and its impact on the workplace.

*“there was definitely guilt from those who went off sick, and when they came back… the others who had dealt with the work… were not welcoming.” (MNG03, Female, Senior Matron, Other Asian)*

## Discussion

In this study, we explored the intersection between work and long COVID for HCWs examining its impact on their professional identity, orientation to work, well-being as professionals, and the support needs and strategies available to them and their managers to continue working. Additionally, we investigated the broader effects of long COVID on HCWs’ home lives, support networks, and healthcare workforce management.

Our findings underscore the challenges faced by HCWs with long COVID, including the disruptions to their professional roles, the varied levels of workplace support, and the emotional burden they experience. These challenges were mirrored by healthcare managers, who struggled to balance workforce demands with providing adequate support, and by HCWs’ support networks, who experienced emotional strain while caring for affected individuals. Overall, the findings highlight the pressing need for standardised workplace policies and targeted managerial training to support HCWs with long COVID while ensuring workforce stability.

We found that long COVID greatly impacted the physical health, home life, and mental well-being of HCWs, with varying levels of resilience and support shaping their ability to cope. These challenges are consistent with those reported in existing literature. Participants frequently described physical symptoms that hindered their ability to manage day-to-day activities, often resulting in significant role changes within their households [21]. Similar findings have been noted in previous qualitative studies, where individuals reported struggling to meet the demands of home life due to persistent symptoms [6]. 

The impact of long COVID on work performance and patterns was profound, with many HCWs expressing their need for flexible working hours or phased returns to work. While positive workplace support was reported by some, others encountered significant barriers, including insufficient accommodations or inadequate recognition of their needs by HR departments. These findings are also supported by existing research studies, indicating the necessity of workplace adaptations for HCWs managing long COVID [22]. However, inconsistent experiences across healthcare settings highlight a lack of standardised support systems, as similarly reported by Gorna et al. [22], who emphasised the need for workplace policies that align with the lived experiences of long COVID patients. 

Additionally, managers and colleagues faced significant challenges in responding to the evolving needs of HCWs with long COVID. Managers described struggling to balance workforce demands with the need to support affected staff, often feeling stressed due to the absence of clear policies and training on long COVID-specific management strategies. Many felt caught between ensuring patient care continuity and providing reasonable accommodations for staff with long COVID. This uncertainty extended to return-to-work processes, with managers expressing frustration over the difficulties in planning work schedules due to the fluctuating severity of symptoms among affected HCWs. Similarly, colleagues reported feeling the strain of covering for long COVID-affected staff, at times expressing frustration at the additional workload while also feeling empathy for their colleagues’ struggles.

The uncertain nature of long COVID further complicated workplace support and return-to-work decisions as the absence of a universally accepted definition or diagnostic criteria for long COVID contributed to some scepticism among colleagues and managers, reinforcing the stigma experienced by affected HCWs.

Additionally, long COVID has led to significant emotional challenges for HCWs, with feelings of guilt and stigma playing a major role. Existing qualitative research also found that stigma associated with long COVID stems from both internally generated shame and externally perceived judgement from colleagues and the broader community [4, 23]. These emotions, influenced by the invisible nature of the condition, complicate workplace dynamics and add pressure on HCWs to justify their illness and prove their need for support.

The findings of this study reinforce the urgent need for clear, standardised policies to address the challenges of long COVID in the healthcare workforce. Current organisational responses remain inconsistent, with many HCWs and managers left to navigate workplace adjustments on case-by-case basis. Implementing formal policies that recognise the fluctuating nature of long COVID and providing structured guidance for phased returns and accommodations would help ensure a more equitable and supportive environment for affected HCWs.

Intervention development should prioritise the integration of standardised, flexible and phased return-to-work policies across NHS trusts, tailored to common symptom profiles. For example, guidance should include structured templates for phased returns, options for reduced or modified duties, and access to quiet spaces or micro-breaks to support those experiencing cognitive symptoms. Embedding these supports into existing systems—such as flexible working and sickness absences toolkits specific for NHS employees with long COVID—would allow for scalable and time-efficient implementation across settings​ [24]. 

Moreover, amendments to NHS absence recording systems and sick pay policies are urgently needed to reflect the episodic nature of long COVID. This includes distinguishing long COVID from acute COVID absences, allowing for intermittent leave without financial penalty, and ensuring long-term absences do not trigger disciplinary procedures automatically. Such changes would alleviate fear and uncertainty among HCWs and support timely access to care and recovery​.

Healthcare managers also require occupational health collaboration and resources to support teams holistically, including funding for temporary cover during long absences and integrating peer support networks to reduce team-level strain. Additionally, they require specific training on managing long COVID-related workforce issues to reduce ambiguity and improve support mechanisms.

While this study focused on HCWs in the UK, the findings carry broader relevance for healthcare systems globally. Beyond the UK context, in resource-limited settings, cost-effective interventions—like peer support groups, simplified occupational health referral pathways, and digital self-assessment tools—may offer scalable ways to support HCWs with long COVID while reducing the burden on frontline managers.

Finally, future research should explore the long-term impact of these workplace interventions on HCW retention, mental health, and productivity. Co-developing interventions with affected HCWs will be key to ensuring feasibility and acceptability in real-world settings. Furthermore, cross-country research collaborations are needed to evaluate the effectiveness of these workplace interventions across diverse healthcare systems and cultural contexts. Recognising long COVID as a global occupational health concern is essential to promote equitable support for the healthcare workforce worldwide.

### Strengths and limitations

This qualitative work has provided a greater understanding of the experiences of HCWs with long COVID from diverse ethnic backgrounds and job roles and the overall impact of long COVID on their home and work lives and the workforce overall including management – an important and under-explored area–. Interviewing three different groups to understand the overall experience and impact was one of its strengths, which provided triangulation for the results. Nonetheless, this research has some limitations worth noting. The sample size, although diverse, may not capture the full range of experiences of HCWs from different healthcare settings or cultural backgrounds. The recruitment strategy, which relied partly on snowball sampling, could have introduced selection bias, as participants might have recommended others with similar experiences.

## Conclusions

The experiences of HCWs managing long COVID are deeply complex, encompassing persistent physical symptoms, altered professional capacities, and significant emotional burdens. The findings highlight a critical need for more tailored and culturally sensitive support systems within healthcare settings. The mixed responses regarding workplace support emphasise the necessity for standardised policies that address both physical and mental health aspects of long COVID, recognising the unique challenges faced by HCWs. Enhanced training for healthcare managers to better understand and support employees with long COVID, as well as further research into culturally diverse coping mechanisms, could improve reporting of long COVID by HCWs from diverse ethnic and cultural backgrounds, and, therefore, improve outcomes for affected HCWs and their support networks.

## Supplementary Information

Supplementary Material 1.

Supplementary Material 2.

## Data Availability

The data for this study consists of interview transcripts of participants that contain potentially identifying and sensitive information. The data cannot be shared publicly due to concerns of participant confidentiality and ethics requirements. Participants consented to the study with the understanding that only de-identified quotations would be made public, not the entirety of the transcripts. Therefore, only illustrative quotes from the transcripts have been included in this article.

## Abbreviations

HCWs: Healthcare Workers
UK: United Kingdom
NHS: National Health Service
HR: Human Resources
PIS: Participant Information Sheet
PEP: Professional Expert Panel
STAG: Stakeholder Advisory Group
PPIE: Patient and Public Involvement and Engagement
NVivo: Software for qualitative data analysis
COREQ: Consolidated Criteria for Reporting Qualitative Research
UK-REACH: United Kingdom Research study into Ethnicity And COVID-19 Outcomes in Healthcare workers

## Footnotes

1 Nurses in the UK at this level typically have more experience and responsibilities than Band 5 nurses, often working as senior staff nurses, specialist nurses, or deputy ward managers.
