# Supplementary material for: The impact of long COVID on UK healthcare workers and their workplace: a qualitative study of healthcare workers with long COVID, their families, colleagues and managers
Source: BMC Health Serv Res. 2025 Apr 9;25:519. doi: 10.1186/s12913-025-12677-x (PMC11980223; doi:10.1186/s12913-025-12677-x)
Supplement: Supplementary file 2 — Supplementary Material 2. [file 12913_2025_12677_MOESM2_ESM.pdf]

Supplementary materials 2

Topic guide for healthcare workers

Topic Guide for healthcare workers (REACH-OUT)

OBJECTIVES
- To gather reflections on participants' views and experiences as clinical and non-clinical healthcare workers post COVID-19;
- To explore healthcare workers' experiences of acute infection (initial COVID-19 illness)
- To examine whether participants have experienced any persistent symptoms/long COVID and what these symptoms have been like over time;
- To discuss the impact of acute and long COVID on mental and physical health. Their work life, provision of care, and their home lives.
- To identify things that have been helpful in their workplace or community in supporting healthcare staff or protecting their health post COVID-19 infection.

- **INTRODUCTION**
  - Introduce myself, project, and funder
  - General informed consent information, e.g., how the data will be used, confidentiality, timing, permission to audio-record and transcribe interview/focus group

- **BACKGROUND INFORMATION**
  - These include basic job-related questions (e.g., work patterns, role in the organisation, part-time/full time).

- **To explore healthcare workers' experiences of acute infection (initial COVID-19 illness)/persistent symptoms (long COVID)**

  1. Tell me about whether you have experienced long Covid, or persistent symptoms after Covid? What symptoms have you had and for how long?
  2. What these symptoms have been like over time?
  3. How are your symptoms now?

- **PERCIEVED FEARS, CONCERNS AND RISK FACTORS OF CONTRACTING LONG COVID**

  1. Did you feel that anything particularly put you at risk of long covid? (both)
  2. What do you think are important risk factors you or other healthcare workers like yourself have for long covid?
  3. Were you ever worried about getting long covid?why?(both)
  4. What are some of the fears or concerns you have post-covid-19? What has influenced these fears?

- **IMPACT ON HOME & WORK LIFE**

  1. Have your symptoms impacted your work life at all? How?

  2. What are your experiences of taking time off sick or changing roles/working flexibly due to long Covid? (*work patterns, sick leaves (what were the implications of that on you? Your colleagues?), financial difficulties, engagement with the job, relationship with colleagues, career progression*
  3. What about your home life? How?
  4. Has it affected your family?
     day to day activities, hobbies, socialising
  5. Has it affected your mental wellbeing? How?

- **ACCESSING/SEEKING HEALTHCARE POST ACUTE INFECTION/LONG COVID**

6. Have you reported your persistent symptoms? To whom? If not, why not?
7. Have you thought to access/seek healthcare for your persistent symptoms? Why/Why not?
8. How did you go about of seeking healthcare? GP referral? Long covid clinic?
9. How has your experience been of getting healthcare for your persistent symptoms? waiting times? How well did it meet your needs?

- **SUPPORT**

1. How much support there have been in your workplace with regards to your post-COVID-19/persistent symptoms?
   - What risk assessments have you had in your organisation related to long covid? What has your experience of them been like?
   - What are your thoughts on the risks or rules you have encountered in your organisation post-COVID-19?
2. What support have you got? And how easy was it to get support? How helpful has it been?
3. Managers? What about Colleagues? How did they support you?
4. Are there any areas where you feel you haven't had enough support?
5. Thinking about your friends, family and community, what about support outside of your workplace with regards to your post-COVID-19/persistent symptoms?

- **POST-COVID-19/LONG COVID STIGMA**

1. Do you have any worries about how other people think of you at your work place? or how you might be treated differently because of long covid? How does that make you feel?
2. Has it been stigmatising? In what way?

- **COPING MECAHNISMS**

1. Thinking about you personally and how you have coped with all of this,
2. What strategies or things you have done to cope with your symptoms?
3. What have you done to cope with how the symptoms of long covid impacted your life

- **KNOWLEDGE ABOUT POST-ACUTE COVID-19 INFECTION/LONG COVID**

1. What do you know about long COVID?
2. What sources of information have you used to know about long COVID?
3. What do you expect will happen to your symptoms in the future? Why? Do you have an idea on how long symptoms would last?

- **PROTECTION AND PROMOTION**

1. Based on your experiences, what do you think would be important to improve the experiences of HCWs with persistent symptoms going forward?
2. What should healthcare organisations do differently to better support HCWs with persistent symptoms? What about your colleagues who are on sick leaves? working extra shifts?
3. What would help in the community to better support HCWs with persistent symptoms?

- **CLOSING**
  - Thank participant for their time
  - Reminder of confidentiality
  - Ask for potential to re-contact if there is there is a follow-up element in the research
  - **Snowball sampling ! Ask for other potential interviewees (i.e., colleagues/household members.**

Topic guide for support network members

Topic Guide for colleagues/managers (REACH-OUT)

**OBJECTIVES**
To explore:<br>• The impact of HCWs' acute infection and long COVID on household members, including home life, mental and physical health and well-being, and wider community and work context;<br>• (If support network members were healthcare colleagues), healthcare colleagues' perceptions around the impact of HCWs' acute infection and long COVID on the workplace, including provision of care, implications for healthcare colleagues' work responsibilities, and the wider work and patient community;<br>• (If support network members were healthcare colleagues), healthcare/occupational changes that have occurred or strategies that have been implemented by healthcare colleagues or the organisation in response to HCWs with acute or long COVID; Key support or coping mechanisms of household members or healthcare colleagues.<br>• Key support or coping mechanisms of support network members.

- **INTRODUCTION**
  - Introduce myself, project, and funder
  - General informed consent information, e.g., how the data will be used, confidentiality, timing, permission to audio-record and transcribe interview

- **BACKGROUND INFORMATION**
  - These include demographic and social factors such as, ethnicity, basic job-related questions (e.g., work patterns, role in the organisation).

- **Healthcare colleagues' perceptions around the impact of HCWs' acute infection and long COVID on the workplace, including provision of care, implications for healthcare colleagues' work responsibilities, and the wider work and patient community;**
  I'm interested in the recovery phase, within the last year or so (after the period of lockdowns)

1. Can you tell me how your organisation has been affected over the last year, in terms of staff experiencing long COVID? What implications has this had for the workforce?
2. What impact has that had on you personally or on your role? Work patterns? Different or new responsibilities?
3. Have you been redeployed to cover the absence of off-sick colleagues? Tell me more about that
4. Have you faced any particular challenges or difficulties due to colleagues experiencing persistent symptoms after acute COVID?
5. How do you feel that affected the provision of care for patients?
6. Has this has any other affects, for example, on Training or quality improvement initiatives?

- **Healthcare/occupational changes that have occurred or strategies that have been implemented by healthcare colleagues or the organisation in response to HCWs with acute or long COVID;**

    1. What strategies has your organisation used to help with workforce shortfalls due to colleague sickness? What do you think about them? What has worked well? What has worked less well?
    2. What support has your organisation provided to work colleagues who have/had long covid/persistent symptoms?
    3. Did you provide any support yourself to colleagues who have/had long covid/persistent symptoms? Can you tell me about that?
    4. Have you experienced additional strain or burden for having colleagues off sick? (emotional burden?)
    5. Have you had support from your organisation for managing the workload, or for your well-being? Do you find it helpful? If not, why? What do you think might be helpful?
    6. Can you tll me more about your experiences of support that the organisation/trust provided for the wellbeing of staff?

    7. **For managers:** Have you had to make decisions about sick leave for staff with long covid? Are there any guidance/regulations that have helped you make these decisions? Were they helpful? What challenges have you faced?
    8. Some HCWs are worried about losing their jobs because of the long sick leave, what do you think about that?
    9. Does your organisation have regulations in place supporting return to work, flexible working etc. for HCWs with long covid? How are you managing that in practice?

- **Coping mechanisms of healthcare colleagues**

    1. Thinking about you personally and how you have coped with all of this,
        a. What strategies or things you have done to cope with the longer term impact of COVID on your workplace?

- **Moving forward**
    2. Moving forward, what can be done better for your colleagues who have long Covid/persistent symptoms from Covid?
    3. What can be done better for you and for colleagues who are working in a workplace where others are suffering from persistent symptoms from Covid?
    4. How can your organisation better plan for the workforce with people having long covid going forward?
    5. To sum up, what would be your top recommendations for supporting healthcare organisations with managing the longer term impacts of Covid on the workforce?

CLOSING
- Thank participant for their time

- Reminder of confidentiality
- Ask for potential to re-contact if there is there is a follow-up element in the research
- **Ask for other potential interviewees (i.e., colleagues/household members.**

Topic Guide for household members/friends (REACH-OUT)

- **INTRODUCTION**
  - Introduce myself, project, and funder
  - General informed consent information, e.g., how the data will be used, confidentiality, timing, permission to audio-record and transcribe interview

- **BACKGROUND INFORMATION**
  - These include demographic and social factors such as, ethnicity, link to the HCW whe interviewed.

- **PERCEPTIONS AND EXPERIENCES AROUND HAVING A friend/HOUSEHOLD MEMBER WITH POST ACUTE INFECTION/LONG COVID**

1. What was your experience of your friend/HM having long covid? Timeline?symptoms?
2. How did you find out? What symptoms did they have?
3. How has that affected you?

- **IMPLICATIONS ON HOME-LIFE**

These include change in day-to-day activity due to physical or mental illness, financial situation, changes in home-life.

1. Have there been any changes to your day-to-day activities due to their long Covid ?
2. Are there things around the house/in their home life that your friend/HM/partner are unable to do or find more difficult to do since they had long covid?
3. How did that affect you?
4. Have you had to take in any extra roles? How do you feel about that?

- **IMPLICATIONS ON WORKPLACE**

These include change in work patterns, change in work responsibilities, and any other occupational changes.

1. Has having someone in your life with long covid impacted your work patterns and responsibilities? In what way?

- ### Helplessness and loneliness/ mental health burden

1. How is it affecting our fried/HM emotionally?
2. How has that impacted you?
3. How much do you feel you can do to help them?
4. How are you feeling? How has that affected your mental health/wellbeing?

- **COPING MECAHNISMS**

1. What strategies or things you have done to cope with having a (person) with long covid?
2. Have you ad any help and support?
3. What are your thoughts about how things might be in the future? Are you making any plans for the future?

- **Financial burden**

1. Has the long Covid had any impact on your financial situation? How?
2. Have you had any support to help with coping financially?

- **MOVING FORWARD**

Moving forward, is there anything that could be done to better support people who are in the same position as you?

1. What can be done better for your friend/HM who have persistent symptoms?
2. To sum up, what would be your top recommendations for supporting healthcare workers with managing the longer term impacts of Covid
3.

CLOSING

- Thank participant for their time
- Reminder of confidentiality
- Ask for potential to re-contact if there is there is a follow-up element in the research
- **Ask for other potential interviewees (i.e., colleagues/household members.**

Topic guide for healthcare/HR managers

Topic Guide for healthcare/HR managers (REACH-OUT)

OBJECTIVES- To explore:

- The personal experiences, challenges, and coping mechanisms of healthcare managers dealing with long COVID/post-COVID effects in their teams, organisations and practices.
- The existing support systems and resources available to healthcare managers dealing with HCWs who have long COVID. This may include examining the effectiveness of organisational policies, employee support programs, and other support mechanisms.
- The strategies and approaches that healthcare managers and HR managers have adopted to tackle the challenges posed by long COVID/post-COVID in their respective healthcare settings.
- Specific training and development need for healthcare managers in relation to long COVID/post-COVID management, team support, and well-being.

Overall, this would contribute to the development of evidence-based policies and best practices to support healthcare managers in dealing with long COVID/post-COVID issues effectively. Additionally, this knowledge would improve support systems, policies, and practices in healthcare settings.

- **INTRODUCTION**
  - Introduce myself, project, and funder
  - General informed consent information, e.g., how the data will be used, confidentiality, timing, permission to audio-record and transcribe interview/focus group

- **BACKGROUND INFORMATION**
  - These include basic job-related questions (e.g., work patterns, role in the organisation, part-time/full time).

- **Experiences and Challenges:**
  - How has your role as an NHS/HR manager been affected by the emergence of long COVID?
  - What is your experience of having employees/HCWs with long Covid within your practice/institution? Tell me more about the impact of this.
  - How do you feel having workers with long covid has impacted on the workforce/led to challenges in staffing more generally?
  - What attitudes have people had about employees with long covid? How do they feel about this?
  - Can you tell me about what adjustments have been made for employees with long Covid? (sick leaves, change in work patterns,..etc)
  - Can you describe any specific challenges you have faced in managing teams due to long COVID?
  - How has this impacted on your role as a manager?

- **Workload and Resource Management:**

- How has the workload for healthcare managers such as yourself changed in the last 2-3 years? How has having HCWs with long covid impacted?

- **Emotional and Psychological Impact:**

  - Have you noticed any changes in your job satisfaction or overall well-being as a result of managing long COVID/post-COVID cases?

- **Organizational Policies and Practices:**

  - Are there any policies in place for supporting HCWs who are experiencing long COVID symptoms? Can you tell me about them? How do you feel about these policies? How did you know about them? Are they easy to access? How did you know about them? Any training?
  - How have the policies regarding long COVID support for HCWs been communicated and implemented within the healthcare setting?
  - In your experience, how well do the existing policies address the unique challenges that healthcare workers with long COVID face?
  - What specific aspects of the current policies do you find particularly helpful and supportive for HCWs dealing with long COVID?
  - How do you think the policies related to long COVID support impact the overall well-being and job satisfaction of HCWs in your organisation?
  - Have there been any instances where HCWs' needs related to long COVID were not adequately addressed by the current policies and approaches? If yes, how were those situations handled?

Moving forward:

- Based on your experiences, are there any aspects of the policies or approaches that you think could be enhanced or revised to better meet the needs of HCWs affected by long COVID?
- What do you personally think should be done to HCWs with long COVID? In terms of sick leaves, work patterns..etc?

CLOSING

- Thank participant for their time
- Reminder of confidentiality
- vouchers
